# Discharge distribution in a multi-outlet spillway with varying adverse conditions

**DOI:** 10.1038/s41598-025-89741-3

**Published:** 2025-02-21

**Authors:** Nils Solheim, Mikael P. A. Hedberg, Gunnar I. J. Hellström, Leif Lia, Anders G. Andersson, Patrik Andreasson, Elena Pummer

**Affiliations:** 1 Norwegian University of Science and Technology, NTNU, Trondheim, Norway; 2 Luleå University of Technology, Luleå, Sweden; 3 Vattenfall Research & Development AB, Älvkarleby, Sweden; 4 Swedish University of Agricultural Sciences, Umeå, Sweden

**Keywords:** Ogee spillway, Discharge coefficient, Spillway capacity, Acoustic Doppler velocimetry, Oblique approach flow, Civil engineering, Energy infrastructure, Hydroelectricity

## Abstract

Hydropower and dam structures worldwide are facing evolving requirements due to changes in climate, better methods for flood estimates, combined with the needs of surrounding interests. Improved understanding of the hydraulic behavior of spillways, and the approach flow leading up to them, is important for evaluation of existing spillways and considering potential redesigns. There is limited research on the distribution of flow across a multiple outlet spillway, therefore a purpose built experimental setup is utilized to examine the impact of various geometrical changes on the flow distribution across a spillway with three outlets. The maximum difference measured between the different outlets were as much as 10%. While small changes to abutment and pier corners were found to reduce total discharge capacity up to 8%, with increased discharge and overflow height causing greater reduction in the capacity of the spillway. To further investigate the flow behavior leading up to the spillway outlets, ADV measurements were conducted to capture flow velocities. The measured flow cross sections indicate a stable flow field leading away from the inlet, stagnation zones and recirculation zones leading up to the spillway, with minor variations occurring for increasing inlet flow rates.

## Introduction

The spillway is an essential component of the dam infrastructure, aptly described by Khatsuria^1^ as the safety valve of the dam-reservoir system. Shifts in weather patterns induced by climate change brings a risk of increasing the frequency and intensity of heavy precipitation events, and hence increasing discharge in most parts of Norway and Sweden^2,3^. In both of these countries, the majority of large dams currently in operation were constructed in the period between 1950 and 1980^4,5^. These aging structures were designed for criteria that were valid at the time of their construction and it is therefore important to understand the effects of increased flow and the impact of adverse conditions.

In the standard design situation, flow is assumed to be uniform and perpendicular to the spillway crest. In reality, approach flow angle might differ from the design conditions, due to irregularities in the riverbed or limitations on the footprint of the structure. This study evaluates the challenge of angled approach flow, combined with higher discharge magnitudes and greater approach velocities, as a significant contributor to adverse conditions affecting hydraulic structures and spillway performance. To assess and quantify these effects, a spillway model with multiple parallel outlets was modified and tested under a range of variable parameters, inspired by design variations observed in existing structures. Additionally, the study investigates the influence of pier and abutment design to better understand their combined effects with oblique approach flow.

Physical modelling has been the standard method for studies of spillway capacity, since their reliability is considered to be better than mathematical predictions, especially in complex flow conditions^6,7^. For the case of a single ogee spillway, a multitude of experimental work has been conducted, such as high head spillway experiments with PTV^8^, theoretical work on modelling spillway crests^9^, and experimental work on sharp and angled corners of a spillway^10^. Additionally, earlier work on the subject includes measurements of pressure distribution along a single ogee spillway^11^ and the capacity of accurate simulations^12^. Other recent work couples experiments to CFD investigations using various turbulence models, but does so for a straight channel with a downstream obstacle^13^. Although cases with more complex geometries are less abundant, nevertheless there are documented cases of small scale models featuring spillways placed beside other inlets, such as turbine inlets^14^, or other spillways^15^. Recently published work on an arced labyrinth weir^16^ presented hydraulic modelling coupled with CFD simulations, where the experimental results deviated from theory. This deviation was assumed to be due to the geometry leading up to the weir obstructing the flow. Older work of similar character shows how CFD can be an important tool in designing ways to increase discharge capacity, as it allows solutions to be tested before a physical model is built for validation^17^. Larger experiments to investigate the interference of multiple separate spillways show that a reduction in spillway capacity occurs when two spillway gates are opened at the same time for the same reservoir, with a key factor being shorter distance between spillways reduced total flow^18^. Previous studies may have investigated the total rating curve or discharge capacity of a spillway with multiple outlets, however the authors present here a unique model which is able to measure the discharge distribution between the various outlets while in normal operation. Some of the previously mentioned cases have a large lateral component in the flow leading up to the spillway. Such lateral approach flow and the general flow conditions are also relevant to studies on contractions and side weirs, akin to the work conducted by Hager^19^. However, the latter focuses on side weirs that do not handle the full discharge. Multiple studies have investigated the discharge capacity of side weirs in various conditions, ranging from prismatic channels and varied weir heights, to converging channels and oblique weirs^19–21^. In terms of contractions in high-velocity flow, further work by Hager^22^ explores the impact of contractions at various angles but does not account for the acceleration of flow that occurs over the spillway.

**Figure 1:**
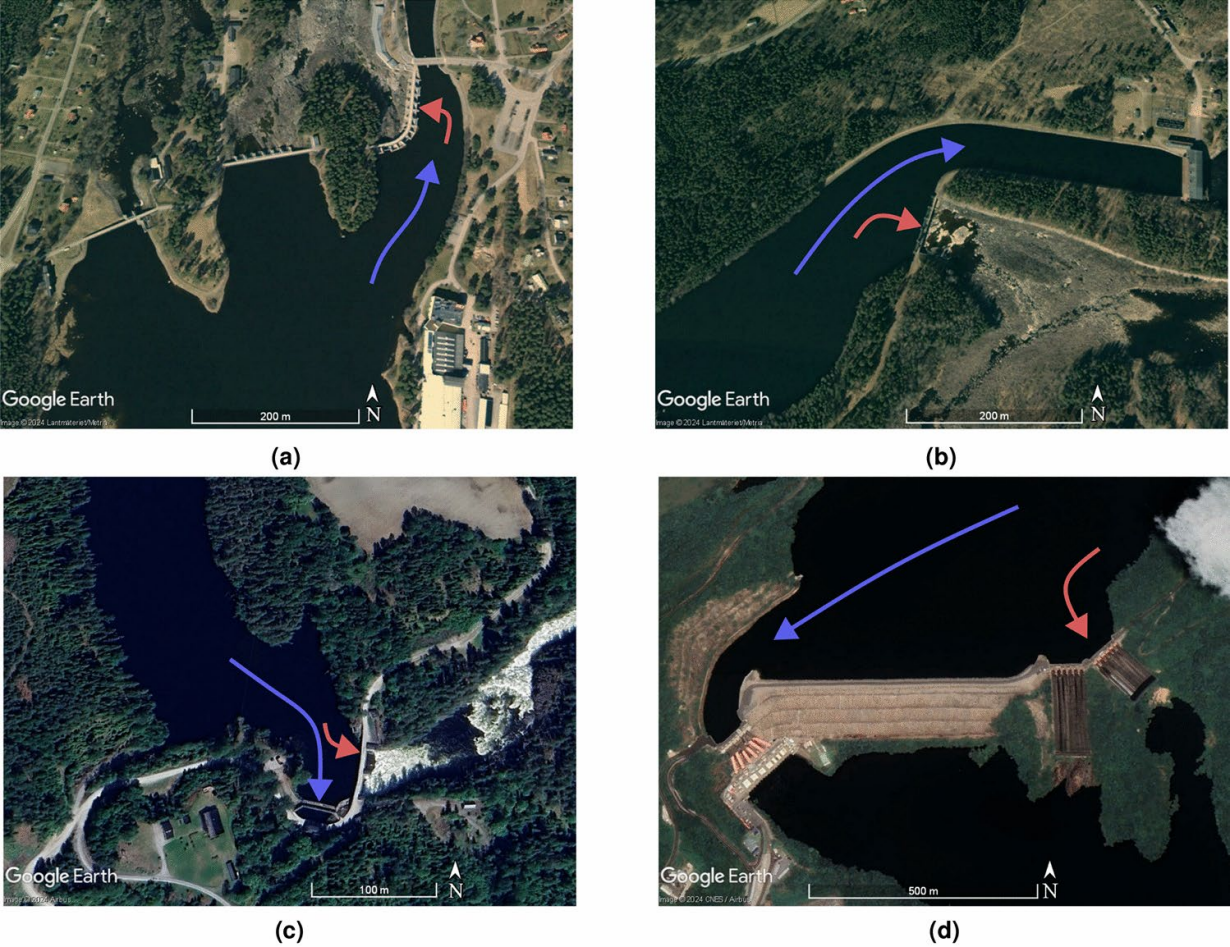
Example cases with blue arrows indicating flow toward the power station, while the red arrows show approach flow toward the spillway. **(a)** Part of spillway axis perpendicular to flow direction toward the turbines in Älvkarleby, Sweden. **(b)** example of spillway axis lateral to flow direction toward the turbines at Lansforsen, Sweden. **(c)** Large contraction on the left abutment with intake placed close to the spillway at Kaggefoss, Norway. **(d)** Spillways with large piers placed on the abutment of the channel in Akosombo, Ghana. Map data provided by Google, Lantmäteriet/Metria, Airbus and CNES/Airbus.

Where avoidable, spillways are typically not constructed near turbine inlets due to potential interference. Something which can be mitigated when inlet structures are constructed in purpose-built channels. For hydraulic structures constructed in natural rivers, common in Norwegian and Swedish hydropower systems, the topography and bathymetry may impose limitations on this design. Figure 1 shows overhead views of dams in Sweden, Norway, and the Akosombo dam in Ghana. In these examples, spillways are positioned either perpendicular or at an oblique angle to the main flow leading toward the turbine intakes. Figure 1 illustrates the main flow direction leading toward the intakes as a blue arrow, while the spillway direction is shown as a red arrow. Existing literature reveals a noticeable knowledge gap in reporting flow distribution across spillways and the effects of adverse conditions on spillway capacity. One aim of this study is to enhance understanding of how poor flow distribution across a spillway impacts its performance and to identify contributing factors to this loss.

This paper presents experiments on an ogee-shaped spillway with three outlets, building on the work of Hedberg et al.^23^ on a similar channel. The novelty of this study lies in exacerbating the adverse conditions by modifying the channel geometry and pier design, resulting in a greater degree of oblique approach and less favourable outflow conditions. These changes, along with the high overflow ratio and increased approach velocity, significantly alter the flow distribution between the outlets and overall spillway performance. This study seeks to investigate how adverse conditions affect discharge capacity, velocity fields and distribution through a multi-outlet spillway.

We hypothesize that the changes introduced to the channel will increase the water level in the channel for comparable flows, indicating a reduced spillway capacity due to the increased oblique angle of the incoming flow. Additionally, straightening the piers and abutments of the outlet will impact the spillway capacity negatively, further reducing the capacity for a given water level. The interactions between these two phenomena causing capacity loss will be quantified in this study. An additional outcome of the unique model study is to provide velocity data for use as boundary conditions and validation data for numerical modelling. The combined sets of experiments can serve as material for evaluating CFD methods and codes for design, redesign and evaluation of hydropower infrastructure.

## Method

**Figure 2:**
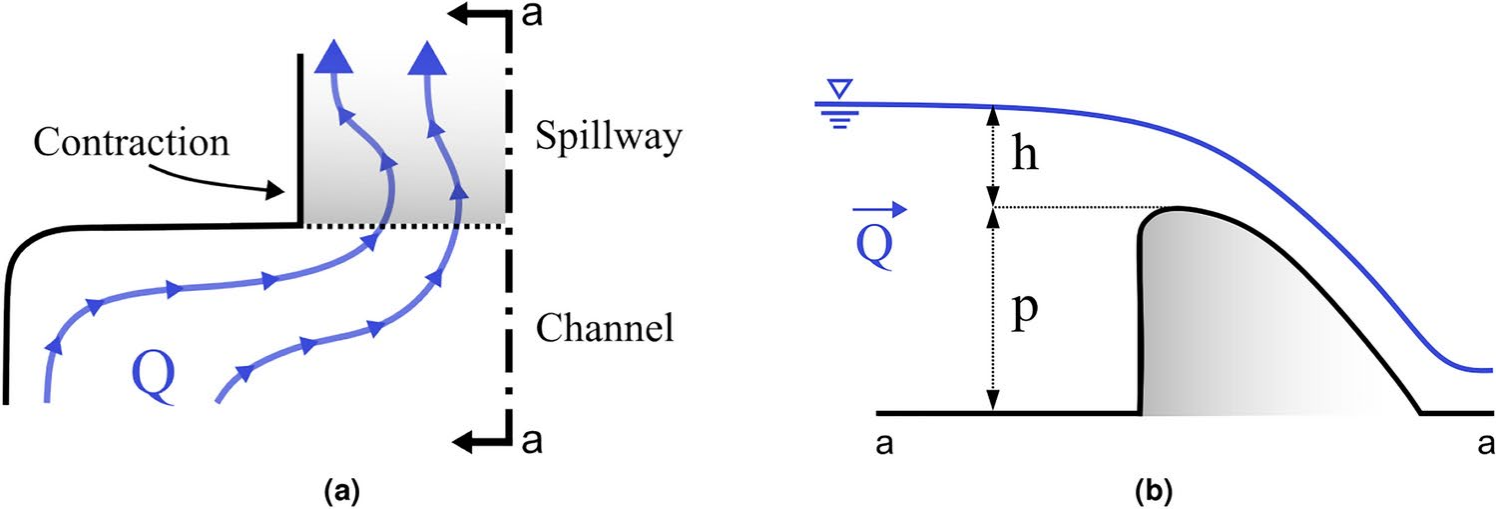
**(a)** Plan view of simple streamlines for a spillway with oblique approach, showing contraction occurring around the abutment corner. **(b)** Section view of a spillway, defining weir height and overflow height.

The discharge over an ogee-crested spillway is given by^24^:

1 $$\begin{aligned} Q=C \, L \, H^{3/2} \end{aligned}$$

where *Q* is the discharge, *C* is the spillway’s discharge coefficient, *L* is the spillway length and *H* is the total head over the spillway crest. To correct for contraction losses occurring at the corner of the abutment and inlet of the spillway, the standard design approach involves taking a reduced effective spillway length, given by: $L_{\text {eff}}=L-(n\, K\, H)$, where *L* is the uncorrected length, *n* is the number of corners and *K* is a contraction loss coefficient. A commonly used contraction coefficient is found in the handbook of Creager and Justin^25^, namely $K=0.1$. The effect is illustrated in Fig.2a, where the full length of the spillway is not utilized due to the sharp corner, which causes a contraction loss. These losses reduce the effective discharge capacity, particularly when multiple corners or obstructions exacerbate the issue. The presence of oblique approach flow further compounds these losses, leading to localized reductions in performance.

Chow^26^ additionally provides summaries of the works by USGS and USACE on topics such as pier design and nose shapes. Further references are made to Yarnell^27^, which compares and expands on key references from the turn of the previous century regarding piers and channel constrictions, including the work by Rehbock, D’Aubuisson, and Weisbach. While the main focus is placed on bridge pier and channel obstructions, the impact of pier geometry and flow angle is briefly examined. This yielded the publications of USACE^28^, which have *K*-values depending on the shape and location of the pier, outlined below.

2 $$\begin{aligned} L_{\text {eff}}=L-2\,(n \, K_p + K_a) \, H \end{aligned}$$

where $K_p$ and $K_a$ are contraction coefficients representing the piers or abutments respectively, with a maximum value of 0.2 found for $K_a$.

A distinction exists in the use of total head (*H*) and overflow height (*h*), with the latter representing the height above the spillway crest and excludes the velocity head component. The relationship between overflow height (*h*) and weir height (*P*), as shown in Fig. 2b, is an important factor for understanding contraction losses and spillway discharge efficiency. The coefficient of discharge (*C*) is influenced by the overflow height, which dictates the recommended curvature of the spillway chute. A higher overflow to weir height ratio (*h*/*P*) indicates increased approach velocities, leading to greater contraction losses. These effects are particularly significant for run-of-river structures with which often feature lower relative weir heights. This study incorporates these corrections into its analysis to better understand the combined impact of oblique approach flow and geometric modifications on spillway discharge capacity. By comparing experimental results with theoretical predictions derived using contraction loss coefficients, this study highlights the limitations of existing design equations and the need for adjustments in design practices for modern hydraulic infrastructure.

### Model configuration

**Figure 3:**
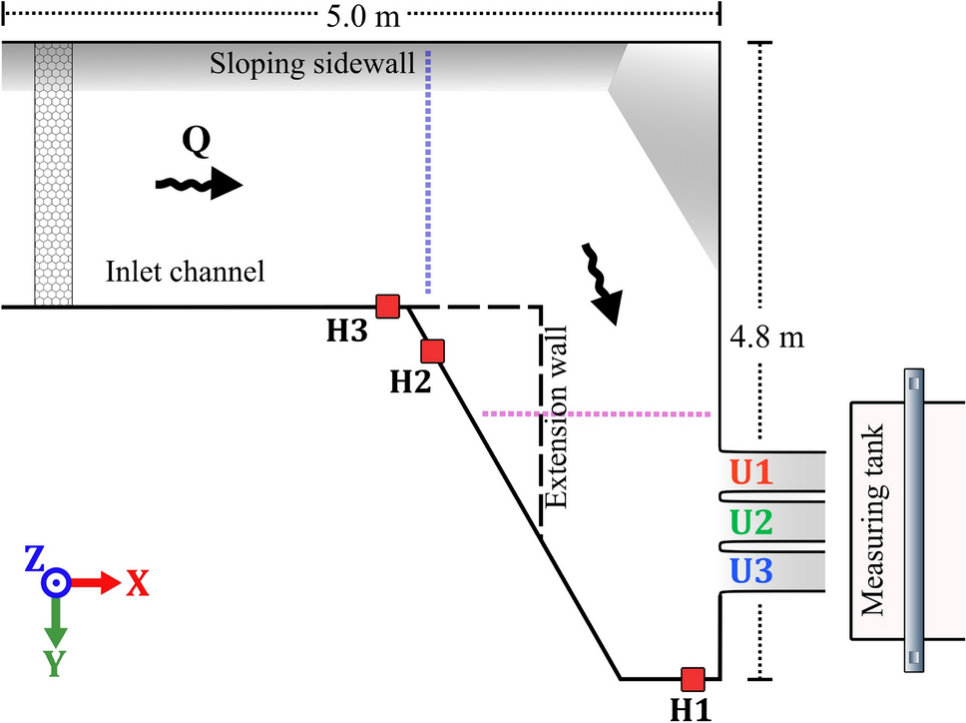
Planar view of experimental setup with ADV positions marked with purple and blue dotted lines at the spillway and in the channel. Honeycomb flow rectifier shown at the upstream end of the channel. Holes for the magnetostrictive sensors shown with red squares.

Experiments were performed at Vattenfall’s hydraulic laboratory in Älvkarleby, Sweden. A schematic overview of the model is presented in Fig. 3. At the inlet, the model is supplied by two pumps with a total capacity of 300 litres per second. Inlet pipes are attached to the model, discharging into a smaller inlet basin before entering the channel. Both inlet pipes are equipped with electromagnetic flow meters for discharge measurement. To ensure an even flow distribution, several plates of perforated steel are positioned near the pipe exits to even out the flow entering the inlet basin. Lastly, a honeycomb mesh leads into the main channel, serving as the final flow-regulating structure and inducing a more uniform turbulence pattern, as these flow characteristics can be utilized as boundary conditions for CFD. The channel is constructed from steel plating with a footprint measuring roughly 5 x 5 m. From the inlet channel, a sharp corner in the model directs the flow obliquely toward the three spillway outlets. The raised floor consisted of steel plates welded onto a steel scaffolding, letting water fill the void under the raised floor. Small gaps in the floor permitted water to trickle up from beneath. Measures were taken to seal the edges and floor close to the outlets. The geometry of the spillway outlets was sourced from the hydropower plant at Torpshammar, Sweden, with the shape being scaled 1:50, manufactured out of polyurethane. Manufacturing defects resulted in slight differences between the three outlets, however the maximum variation was found to be less than 3 mm, within 0.1$\%$ of the intended width of 300 mm. To monitor the water level in the channel, water is diverted through tubing into separate magnetostrictive sensors (see Fig. 3 for positions of the sensors).

The flow measurement for a specific spillway outlet was accomplished by diverting the flow from the spillway chute into a 6 m$^3$ tank suspended on four load cells. Internal testing of the load cells at Vattenfall’s concrete laboratory, showed an uncertainty of ± 0.1%. Sensor drift was compensated for in post-processing by calibrating the measurements after blocking two of the three outlets. The load cell data, along with readings from the magnetostrictive sensors and volumetric flow rate from the pumps were recorded with the software Labview from National Instruments. As the recording captures data before and after water is diverted into the measuring tank, an interval of at least 30 seconds of flow was taken for data analysis; when possible, more time was used. This limitation comes as a result of the volume constraint of the weighing tank, as larger discharges filled the tank at an increased rate. The mean measurement time across all experiments was 70 seconds. Comparison between short and long time-series showed differences below the 1% uncertainty of the measurement instruments.

The load cell output is plotted against time, which yields a slope that provides an accurate measurement of discharge through the individual spillway outlet. This is repeated for all three spillway outlets with constant inflow to the model. The sum of measured outflow through the spillway outlet can be compared to the incoming flow measured from the pumps, giving a percentage of the total flow which has passed through each individual outlet. Calibration was conducted by blocking two outlets and adjusting the signal from the weight tank to match inlet flow. Data were gathered for each individual outlet, then repeated for that specific inflow three times, this was repeated over three days, providing a total of nine sets of data for each inflow. During measurements there was slight leakage between the outlet and weighing tank, estimated to be less than 0.1 l/s.

**Figure 4:**
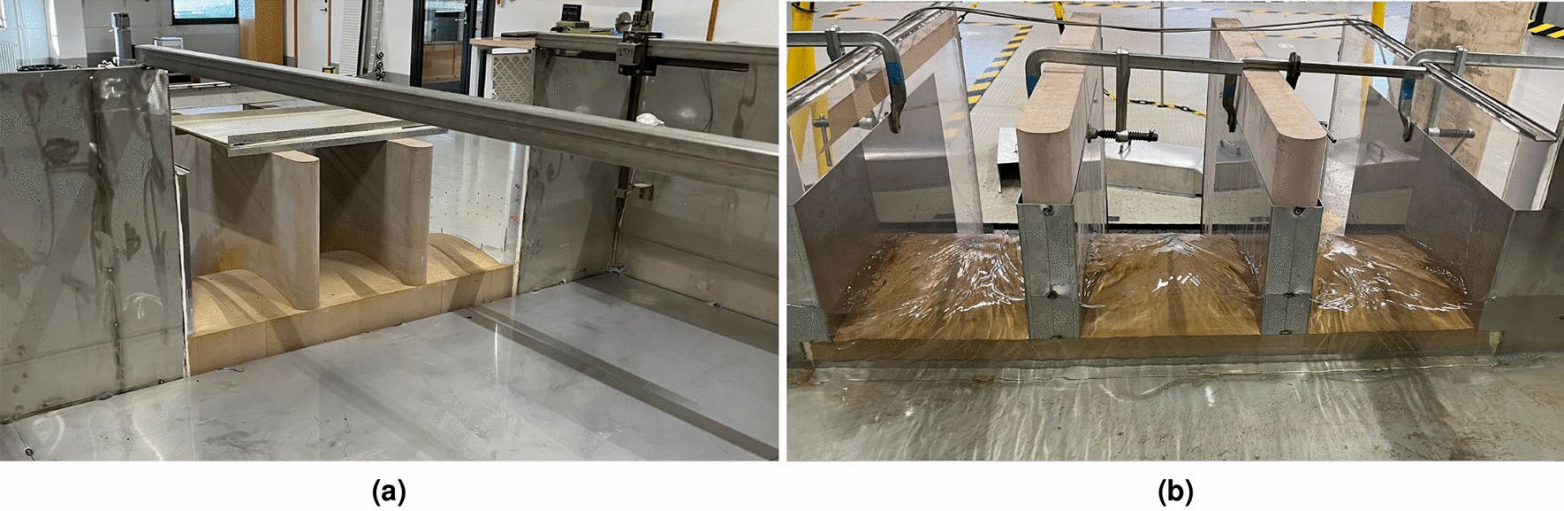
**(a)** Original configuration of outlet with rounded piers shown without water. **(b)** Outlet with corner profile configuration shown for a low flow case.

The initial design, termed the *original* configuration, incorporated rounded corners with a 40 mm radius at the abutments, and a 38 mm radius for the piers, as depicted in Fig. 4a. To investigate the effects of corner geometry modifications, straight aluminum plates with a thickness of 1.5 mm were attached to the spillway piers and abutments, creating sharp corners at the spillway inlets, as shown in Fig. 4b. The model was further adapted by modifying the channel geometry to include an extension wall, increasing the degree of lateral approach flow. The extension wall is marked with a dashed line and in Fig. 3. Model configurations incorporating this customization were designated as *with extension wall*, while those without were termed *without extension wall*.

By combining Eqs. (1) and (2) an estimate of expected increase in water level needed to discharge the same amount of water can be calculated. *L* in the formula for this case would be modified by -9 mm, corresponding to the change in total width, *H* would be the corresponding *H* for $Q=90$ l/s.

3 $$\begin{aligned} \left( \frac{Q}{C\,(L-2(n \, K_p+K_a)\, H)}\right) ^{2/3}=H_{new} \end{aligned}$$

Using two different values for *L*, one as 900 mm, the original width, and second value of 891 mm, for the reduced width by $-9$ mm. Applying Eq. (3) results in a difference in $H_{new}$ of 1.1 mm, indicating any differences in water level greater than 1.1 mm is not caused by the reduced width of the spillway. It is important to note that for calculation of the discharge coefficient the actual length is used, and thus the reduction caused by the corner profiles are included in the comparison of Fig. 6.

To capture the flow field at relevant points in the channel, Acoustic Doppler Velocimetry (ADV) was chosen as a tool. ADV has seen rigorous use in the literature, with some examples including Jeon et al.^29^, and Pandey et al.^30^, where ADV was used in smaller channels to record flow velocities around submerged obstacles. The specific ADV instrument utilized in this study was the Nortek 10 MHz Velocimeter. Data was recorded over one minute with a recording frequency of 100 Hz. The time of the ADV recordings were chosen as a compromise based on the data needed to get good average values and was deemed long enough to yield usable flow velocity values, balanced against the amount of data points that needed to be gathered. In the literature, the time used for recording with ADV varies, Jeon et al.^29^, used 5 minutes, Pandey et al.^30^ used 3 minutes, while Singh et al.^31^ used 4 minutes. Other recent works used as little as 30 seconds of recordings for gathering average velocities with a frequency of 50 Hz, such as Son et al.^32^, while Kumar et al.^33^ with the same frequency made recordings for 1 minute. The gathered ADV data was post-processed in MATLAB according to specifications from the manufacturer for average velocities, filtering the data based on a signal-to-noise ratio (SNR) of 5 and correlation of 70. Since the ADV probe must be inserted into the flow, it is considered an intrusive method. However, the system requires no laser and usually no artificial seeding. Sufficient particulate matter was suspended in the water, eliminating the need for artificial seeding to obtain quality data. Limitations were observed near the sloping side wall, where the proximity of the bed limits measurement as reflections from the sloped wall caused significant interference leading to poor quality data. Similarly, data gathered close to the inlet was examined but deemed unusable due to high variability in velocities and proximity to inlet and bottom, which contributed to the low quality. ADV measurements were taken at two sections within the channel. Along these two lines a grid of points were recorded in intervals of 10 cm in the direction perpendicular to the wall connected to the spillway, and 5 cm intervals in the z-direction. The amount of grid points recorded depended on water level as increased flow allowed an additional point to be measured. The first measurement location was located immediately after the corner leading toward the outlets, as indicated by a blue line in Fig. 3. The second location, marked by the purple line in Fig. 3, was located at a distance of 30 cm upstream of the spillway.

## Results

### Spillway capacity

**Figure 5:**
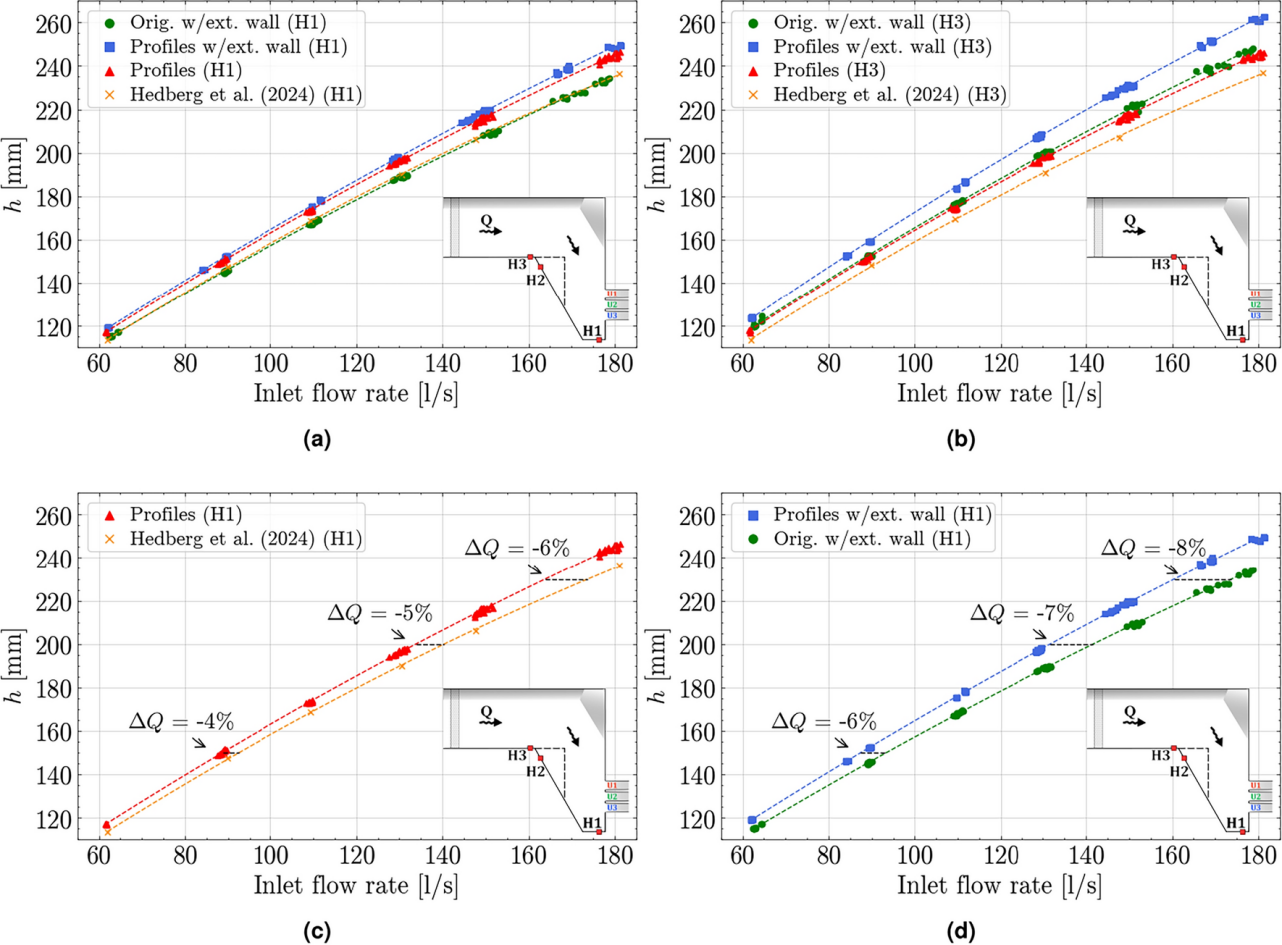
Water level as a function of discharge for the various configurations. **(a)** showing water levels measured at the stagnation point for all configurations. **(b)** showing water level measured upstream for all configurations. **(c)** focuses on the capacity change seen for configurations featuring no extension wall, while **(d)** show capacity-changes with extension wall. Annotations shows the decrease in discharge capacity for a given water level based on the polynomial regression lines.

To evaluate the influence of the various model configurations, Fig. 5 displays the water level measured at the stagnation zone (H1) and upstream by the inlet channel (H3), for the range of tested discharges. Water level (*h*) is plotted in mm over the crest of the spillway against the inlet flow rate (l/s). Each measurement is presented as a scatter plot, accompanied by a second-order polynomial trendline. Figure 5a displays the water levels measured at H1 in the stagnation zone. No discernible difference is observed between the original outlet configuration with extension wall and the results of Hedberg et al.^23^, indicating that the extension wall only increases the water level upstream of the outlet section. Figure 5b highlights the largest discrepancy occurring between the model configuration including both extension wall and the outlet profiles and Hedberg et al.^23^. An element of both reduced outlet capacity, and added degree of oblique approach caused by the extension wall. In this result, both configurations featuring the extension wall display higher water levels than their counterparts without extension wall, related to the added headloss occurring around the sharp angle of the extension wall. Figure 5c, d display the measured H1 water levels for the two configurations, separated into *with* and *without* the extension wall. These figures reveal the variation in discharge caused by the outlet profiles with annotations displaying the difference in discharge capacity between the compared configurations for a given water level.

For the configurations without the extension wall (Fig. 5c), and the given water levels $h=[150,200,230]$ mm, including the outlet corner profiles cause a 4, 5 and 6 % reduction in discharge. Whereas for the configurations including the extension wall (Fig. 5d), the reduction in discharge due to the corner profiles are 6, 7 and 8 %, indicating that the loss of capacity due to the shape of the piers are more significant for the more lateral approach flow. Summarizing the result, the changes made to the model induce greater losses, which results in a greater driving head needed to pass the equivalent water over the spillway.

**Figure 6:**
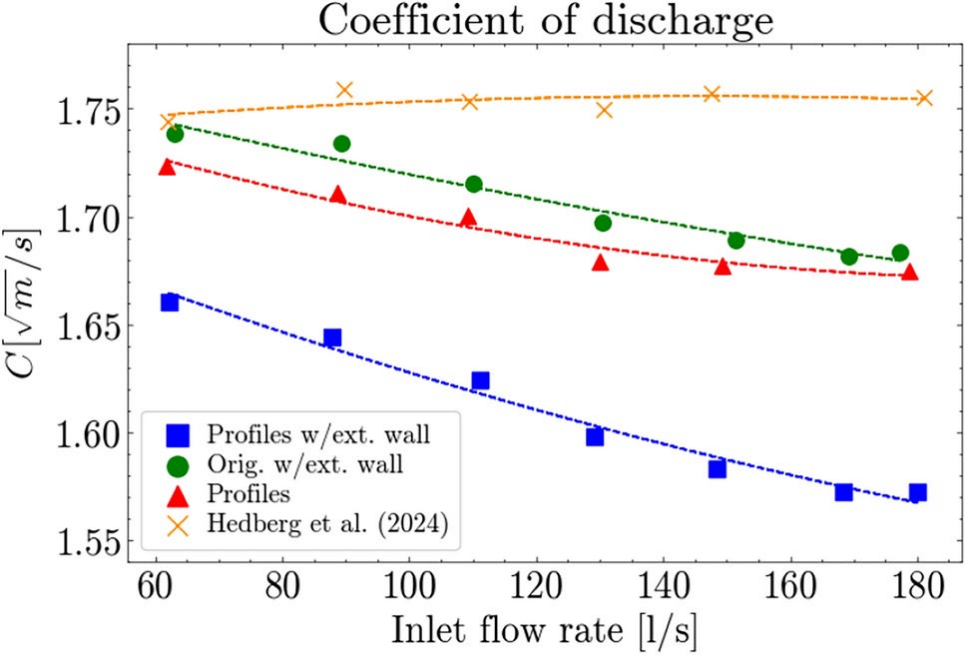
Coefficient of discharge for full spillway, using an average of H1 and H3.

The stage-discharge plots (Fig. 5), do not consider the loss of spillway length, as the corner profiles have a thickness of 1.5 mm narrowing the total spillway length by 9 mm, equivalent to a 1 % reduction in crest length. This consideration is included in the calculated C-factor which is presented below. Equation (3) provides an estimate of water level difference caused by reduced width of the spillway and the results presented in Fig. 5 show differences greater than the 1.1 mm induced by the change in width. The efficiency of the various configurations can be compared by investigating the coefficient of discharge for the total spillway, as outlined in Eq. (1). Figure 6 computes a C-factor for each configuration across the selected discharge levels using a mean water level of H1 and H3. The figure shows a drop in C-values with increasing discharge, for the configurations containing either corner profiles or the extension wall, while Hedberg et al.^23^ maintains a stable coefficient across the discharge levels.

The discharge coefficient provides a metric for comparison with other experimental work and design recommendations, as well as normalizing the discharge capacity between the model configurations conducted in this study. In this result, as spillway length is accounted for, the detrimental effect of increased oblique approach and sharp-cornered abutments and piers have similar effect on the discharge coefficient. Whereas the combined effects of these modifications exacerbate the losses severely.

**Figure 7:**
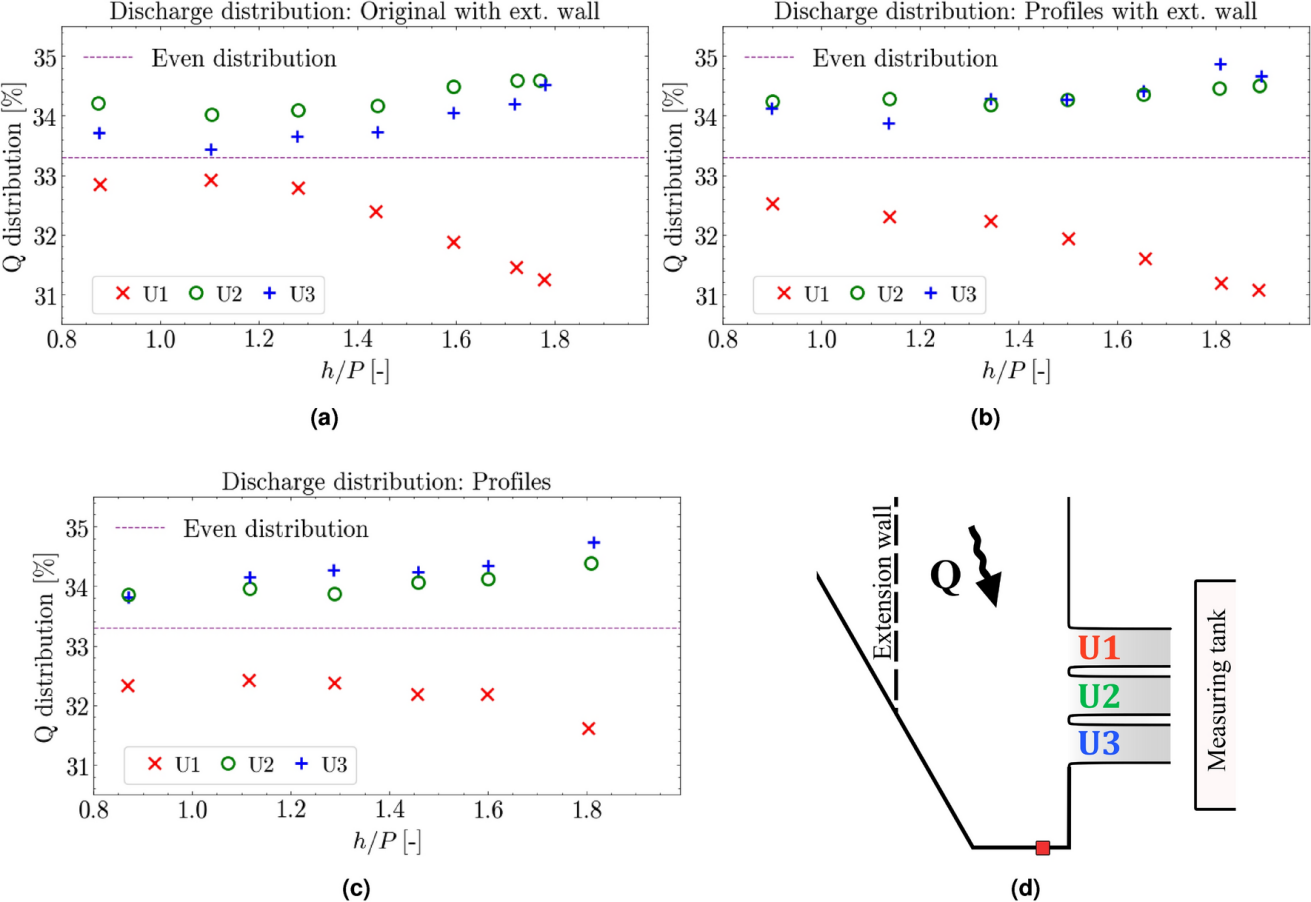
Discharge distribution ($\%$) between the outlets for the various configurations shown for the dimensionless overflow height (*h*/*P*): **(a)** original configuration with extension wall, **(b)** profiles with extension wall, and **(c)** the configuration of profiles without extension wall. Additionally, **(d)** presents a planar overview of the setup and different spillway outlets.

### Discharge distribution

A unique aspect of the model in this study is the ability to measure and compare the performance of individual outlets of the spillway during operation. Evaluating the discharge distribution between the model configurations provides insight into the effect of the overflow height ratio and oblique approach. Presented in Fig. 7 are the results for the distribution of discharge between the three outlet gates displayed as a percentage of the total incoming flow volume as a function of the dimensionless overflow height to weir height ratio (*h*/*P*).

For all configurations, discharge through the outlets are unevenly distributed. Increasing discharge and the overflow height ratio causes the distribution to diverge between U1 and the remaining two outlets, U2 and U3. At $Q_{max}=180$ l/s, the highest overflow ratio occurs, and the most skewed distribution is seen. The largest discrepancy between outlets is seen for the configurations featuring outlet corner profiles and the extension wall, yielding a minimum of 31.0 % for U1 and a maximum of 35.1 % for U3. The original configuration with extension wall (Fig. 7a) shows the tightest grouping between the three outlets until the overflow height ratio increases to $h/P > 1.4$, where there is a sharp decline in outlet discharge for U1. Additionally, the middle outlet, U2, sees a higher percentage of flow compared to U3 for all overflow height ratios. For both configurations containing the corner profiles at the entry to the chute (Fig. 7b, c), U2 and U3 are grouped closer together with U3 showing a minor but consistently higher amount of flow for the case seen in Fig. 7b. For a total printed overview of results, see Table 1, 2, and 3.

As shown in Fig. 7, the discharge distribution between outlets U2 and U3 follow each other rather closely across all measurements. For the configuration with only corner profiles (Fig. 7b), U1 has a stable flow distribution at low discharge, yet the distribution reduces with increased discharge. This can be explained by the increasing overflow ratio, as higher discharge with minor increase in water level yields higher velocities. Which, in turn forces water past the U1 outlet and more towards U2 and U3.

Comparing water levels with only the profiles shows a low impact of increased discharge, as seen in Table 3, where H1 and H3 differ by only a millimeter. Comparing this to Hedberg et al.^34^, which reports similar data without the extension wall or corner profiles, shows that without the profiles the differences between H1 and H3 increase with inlet flows of 90 and 130 l/s, although not at the same rate as when the extension wall is added. For the case of both profiles and extension wall, a combined behavior is observed. The differences gradually increase, and the differences between U2 and U3 remain low. This results in a relatively increased flow through U3, evening out the flow differences compared to the case without corner profiles.

**Table 1:** Table of values for original abutments and pillars with extension wall. Corresponds to Fig. 7a.

Mean inflow [l/s]	Mean out1 [%]	Mean out2 [%]	Mean out3 [%]	H1 [mm]	H2 [mm]	H3 [mm]	$\Sigma$ ratios [%]
63.1	32.85	34.20	33.71	115.6	117.0	120.9	100.76
89.3	32.91	34.01	33.43	145.3	147.9	152.5	100.35
110.2	32.39	34.09	33.64	168.2	171.6	177.1	100.12
130.4	32.39	34.16	33.72	188.9	193.3	199.7	100.27
151.3	31.87	34.48	34.05	209.2	213.8	221.6	100.4
177.3	31.24	34.58	34.5	233.0	238.7	246.6	100.32

**Table 2:** Table of values for profiled abutments and pillars with extension wall. Corresponds to Fig. 7b.

Mean inflow [l/s]	Mean out1 [%]	Mean out2 [%]	Mean out3 [%]	H1 [mm]	H2 [mm]	H3 [mm]	$\Sigma$ ratios [%]
62.1	32.53	34.23	34.12	119.2	120.5	123.8	100.88
87.8	32.30	34.28	33.87	150.2	152.6	156.9	100.45
111.0	32.22	34.17	34.28	177.1	180.3	185.6	100.67
129.1	31.93	34.26	34.27	197.2	201.2	207.6	100.46
148.3	31.60	34.36	34.42	217.6	222.0	229.1	100.38
180.1	31.06	34.50	34.66	248.7	253.6	261.5	100.22

**Table 3:** Table of values for profiled abutments and pillars without extension wall. Corresponds to Fig. 7c.

Mean inflow [l/s]	Mean out1 [%]	Mean out2 [%]	Mean out3 [%]	H1 [mm]	H2 [mm]	H3 [mm]	$\Sigma$ ratios [%]
61.7	32.33	33.85	33.81	117.1	116.8	117.8	99.99
88.7	32.41	33.96	34.14	150.1	149.2	151.3	100.51
109.2	32.38	33.87	34.26	173.3	171.7	174.4	100.51
129.9	32.19	34.06	34.24	196.2	194.2	197.3	100.49
149.2	32.18	34.11	34.34	215.4	213.2	216.6	100.63
178.8	31.61	34.37	34.74	244.0	241.0	244.4	100.72

### ADV measurements of approach flow

**Figure 8:**
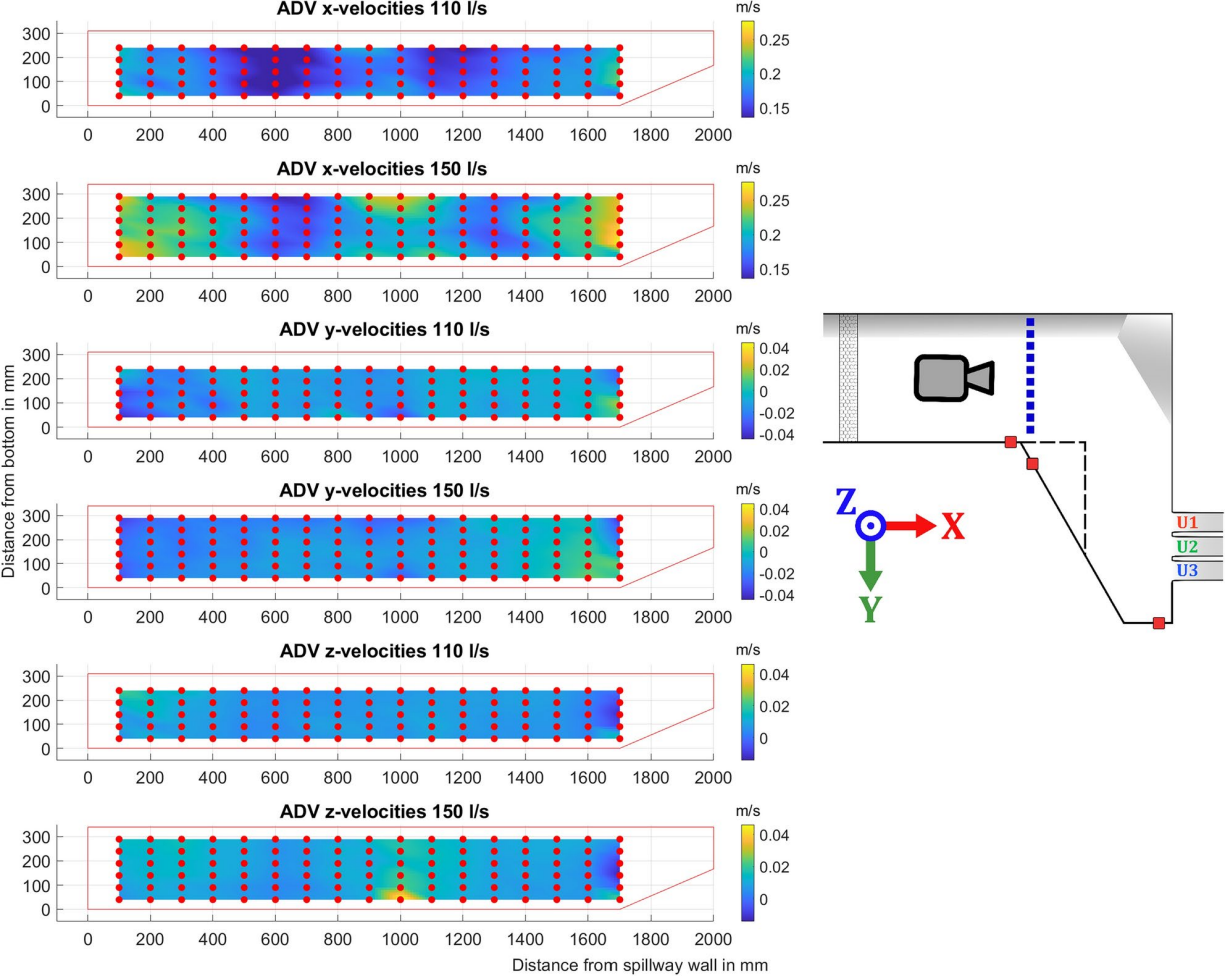
ADV data for the velocities in the three directions, red dots show the location of the ADV point measurements. The red outline indicates the approximate boundaries and water level of the channel. Included is a miniature figure of the setup shown in Fig. 3 with coordinate system, camera icon representing point of view, and a dashed blue line marking the location of measurements.

Two sections of the channel were documented by ADV measurements. One cross section was taken at the beginning of the extension wall, marked in blue in Fig. 3. The second cross section was taken 0.3 m upstream of the spillway outlets, marked in purple in Fig. 3. In Fig. 8 the velocities in the x-direction are shown for two different discharge levels, 110 l/s and 150 l/s. As seen in Fig. 8, the whole channel is not captured. Close to the wall with the sloping floor the ADV picks up interference from reflections off the steel bed resulting in poor quality data. In Fig. 8, for the z-velocities, a large increase is recorded in the velocities close to the bottom, this is likely due to a small positioning error as the probe was placed above a gap in the steel plates. This resulted in significantly larger velocities being recorded in one point. Due to this phenomenon not being recorded for all flow volumes it was deemed negligible. This phenomenon also prevents the ADV from being used too close to the side wall, a choice was made to have the first point being captured at 10 cm out from the wall where good quality data could be ensured.

Due to the inlet design, the flow is not completely reminiscent of fully developed open channel flow, where velocities would be lower at the edges and concentrated in the middle. The distribution is consistent for both the examined discharge levels. Previous work indicates that the impact of inlet velocities shows little effect on the distribution of flow across a spillway for a similar setup^23^. An average of the data gathered in this plane, documented in Fig. 8, can be taken as the average velocity over the inlet. Combining this with the area based on the recorded water level and boundaries of the flume before the corner by the first pressure sensor (H3), the volumetric flow rate can be calculated ($Q=V\,A$) to estimate how well the measured velocities capture the total flow. The resulting values for the 110 l/s case being: $Q=100.5$ l/s, and for the 150 l/s case being $Q=136.3$ l/s. As these values show a deviation of close to 10%, an assumption supported by these measurements can be made that around the edges, where the ADV has trouble capturing quality data there is more flow and higher velocities than the average captured by the ADV.

**Figure 9:**
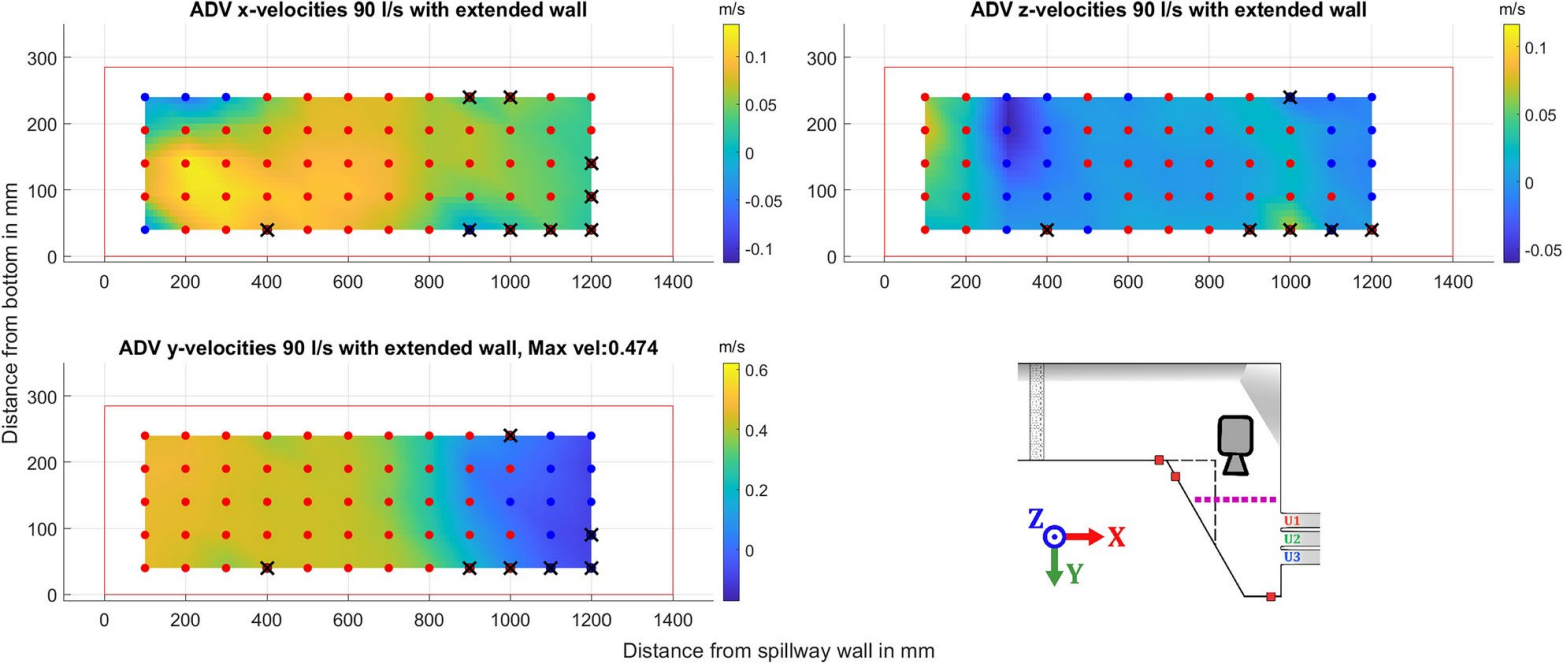
ADV data for the different components recorded for a flow of 90 l/s with an illustration of the geometry and where the data was gathered shown with a dotted purple line, a dashed black line represents the extension wall. The dots show the approximate grid where the ADV data was gathered, color indicates positive or negative mean velocity according to the coordinate system shown in the figure, with blue for negative, and red for positive. Black crosses indicate low quality of the gathered data. Camera icon in the figure shows point of view for the ADV data.

**Figure 10:**
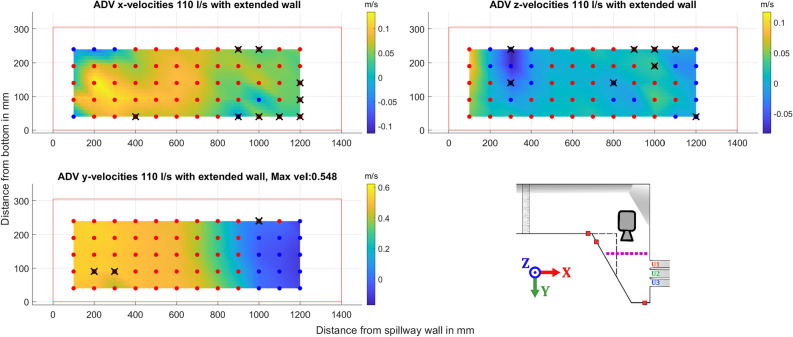
ADV data for the different components recorded for a flow of 110 l/s with an illustration of the geometry and where the data was gathered shown with a dotted purple line, a dashed black line represents the extension wall. The dots show the approximate grid where the ADV data was gathered, color indicates positive or negative mean velocity according to the coordinate system shown in the figure, with blue for negative, and red for positive. Black crosses indicate low quality of the gathered data. Camera icon in the figure shows point of view for the ADV data.

**Figure 11:**
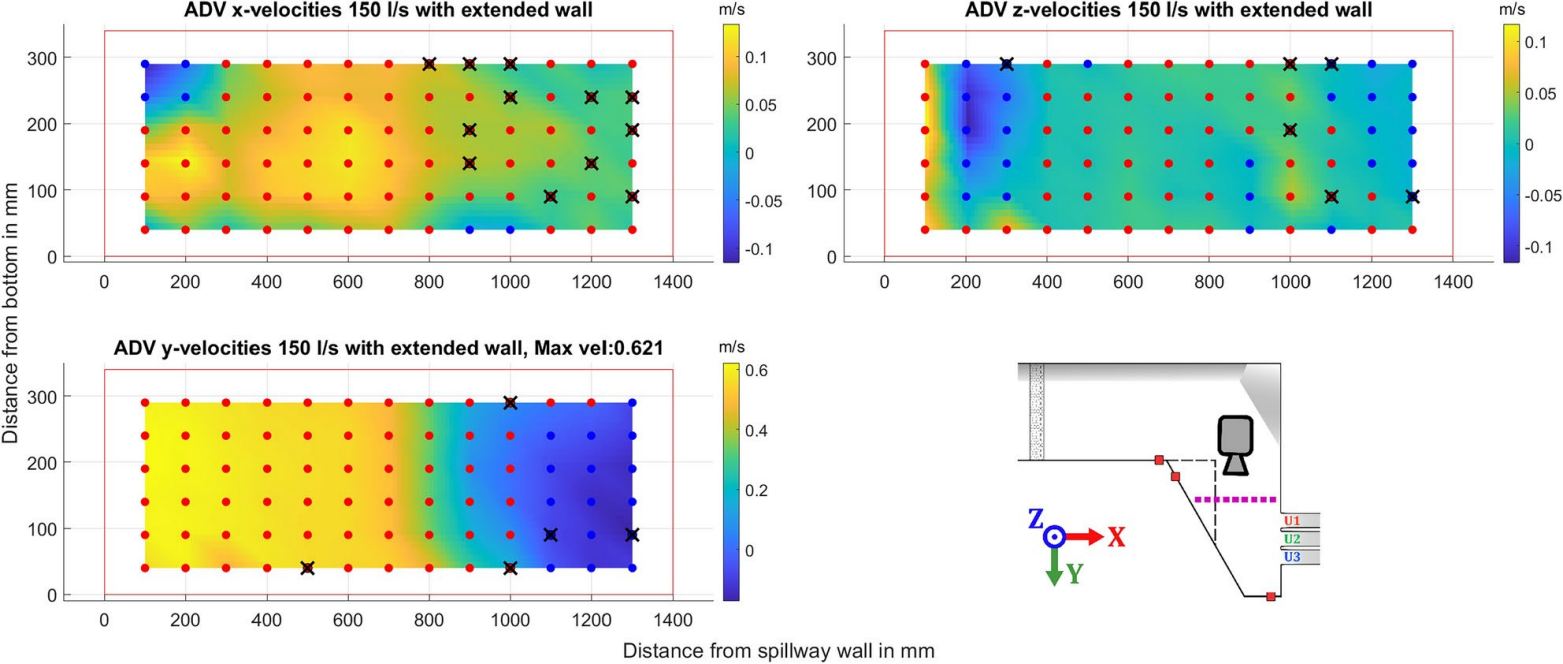
ADV data for the different components recorded for a flow of 150 l/s with an illustration of the geometry and where the data was gathered shown with a dotted purple line, a dashed black line represents the extension wall. The dots show the approximate grid where the ADV data was gathered, color indicates positive or negative mean velocity according to the coordinate system shown in the figure, with blue for negative, and red for positive. Black crosses indicates low quality of the gathered data. Camera icon in the figure shows point of view for the ADV data.

The second cross-section presented is close to the spillway, marked in purple. The ADV data shows similar results for all three cases presented, in Figs. 9, 10, and 11 respectively. All three flow cases show a clear recirculation zone in the x-y plane near the extended wall. The largest recorded zone was for the discharge of 110 l/s beginning at 1000 mm, while both the 90 l/s and 150 l/s the zone seems to start closer to 1100 mm. Close to the wall connected to the spillway there is also circulation in the x-z plane as water is pushed up against the wall and then moves back into the channel. This occur for both flows explored, with the circulating flow seemingly being more prominent for the higher flow case in the x-z plane. Points where large amounts of data were filtered, greater than 20% of points, are marked with a black X. The reasons for the poorer quality data at some of the points could be related to the proximity to the floor and water surface. Some differences can be seen in the higher flow case of 150 l/s as the higher water level brought by the increased flow allows an additional row of data points to be gathered. As the movement in the x-direction of the water close to the spillway wall is a lot clearer with another point close to the surface.

## Discussion

The extension wall significantly increases flow velocities near the outlets, when comparing the ADV data in Figs. 10 and 11 with so far unpublished ADV data. Maximum velocities increased by 49% and 40%, respectively, notably without raising the water level at H1 (Fig. 5a). The oblique approach angle and increased velocity appears to cause water to bypass U1 and increase discharge through U2. Straight corner profiles have a lesser effect on the diverging discharge distribution. Even at low flows, the distribution is uneven, and the increase in distribution difference with higher flows is minimal, except at the highest discharge levels.

The discharge coefficient remains stable for the original design of the channel, shown in Fig. 6. For all the altered configurations, the discharge coefficient drops with increasing flow and *h*/*P* ratio. Increasing water level beyond the design head is expected to yield an increase in discharge coefficient as sub-atmospheric pressure conditions occur over the crest^28,35,36^. This can also be seen in recent experiments by Lou et al.^37^, who performed experiments with flow over an ogee spillway with head ratios above design head, for gated and ungated flow, where they recorded an increased discharge coefficient. In the altered configurations tests, the opposite occurs, indicating losses occurring at the pier and abutment are greater than the increase in discharge coefficient caused by the larger pressure differential over the spillway. The trend appears to taper off at the three highest discharge levels. On the other hand, for the base case (no extension wall, no corner profiles) the coefficient remains stable. This may be of relevance for rivers with high sedimentation as buildup causes an increased *h*/*P* ratio, leading to a lower discharge coefficient and a reduced discharge capacity. Comparing this by the work of Lee et al.^18^, where they concluded that the proximity of multiple spillways was the key factor of reduced discharge capacity when operated simultaneously. In the works of Martinerie et al.^15^, one of the more similar studies to this work, with multiple outlet spillways located roughly 45$^{\,\circ }$ from the main approach channel. They quantified stage-discharge curves, however they merely estimate the changes in *K* representing the pier and guide wall designs.

Seen in Table 1, 2, and 3, the summed discharge ratios are slightly greater than 100%, attributed to several factors. Firstly, drift in the sensors was found to occur over time, noticed during earlier work with the same equipment done by Hedberg et al.^38^. Calibration of the sensors were conducted prior to the experiments in this study, and evaluation in post-processing showed a slight drift of the sensors leaving the calibration slightly outdated. Quantifying the contribution to the overall ratio of the calibration error as less than 0.5%. Due to the results being compared for relative changes, this drift was not corrected for by some correction factor which would require further assumptions to be made. Additionally, the diversion bucket used to separate flow into the measurement tank could leech water from the outlets not in use. Friction from the tight seal could lead to parts of the seal being torn, leading to leakage. Such leakage, if substantial enough to surpass the error range of the instruments ± 1%, would be very noticeable when measuring is in progress, leading to an aborted attempt and seal being repaired. Small, but visually noticeable leakage around the outlets were controlled and estimated through volumetric measurement to be below 0.1% of the inflow.

ADV data of the water moving towards the spillway as shown in Figs. 9, 10, and 11 show steady distribution in the lateral direction, as an increase in flow only showed relative increases in velocities while the recirculating behavior start around the same point in the x-direction away from the spillway. In the y- and z-directions there is an area close to the spillway adjacent wall that, as water is pushed towards the wall it moves upwards creating what seems to be a rotating flow. This area seems to be pushed closer to the wall for the 150 l/s case but due to the distance between measurement points this will remain uncertain. These two zones captured by the ADV provides good verification data as a good simulation should recreate these flow phenomena and may be able to find a relation between size of recirculation zone and flow amount. For comparison with CFD, the jump between data points is quite large at 100 mm and 50 mm respectively, which may provide a misleading view of where the boundary of the recirculation zone lies. The smaller recirculation zone for 110 l/s could be correlated with the flow distribution behavior recorded at 110 l/s as it shows the least variation between the outlets seen in 7a, although limited to one of the cases. The amount of data gathered in each point is low for an ADV measurement and should not be used to evaluate TKE numbers.

## Conclusion

Disturbances to the flow leading up to a spillway can significantly decrease discharge capacity. Laboratory experiments were conducted for three separate setups to investigate the adverse conditions of high overflow ratios and oblique approach flow, including measurement of the discharge distribution through the separate spillway outlets. Modification of the model included changing the rounded abutments and piers to sharp corners. Secondly, narrowing the channel leading up to the spillway and increasing the oblique angle of approach. The third configuration combined these changes introduced in both setups.

Evaluating the rating curves (Fig. 5), show maximum water levels occurring for the model configurations including the extension wall. For a given water level, the results show that straightened abutment- and pier corners yielded a 6% reduced spillway capacity. The configuration featuring both sharp corners and extension wall reduced the total discharge capacity by 8%. Summarized, this entails that the increased oblique approach angle yields a greater loss to spillway capacity. Ultimately, this finding can serve to emphasize the importance of approach flow angle for engineers designing future spillways.

Increased discharge had the largest impact on flow distribution between the outlets, for the case without corner profiles. For low flow rates, the smallest deviations were found, even contracting slightly from the 60 l/s case to the 90 l/s case. The total change in recorded mean flow from 60 l/s to 180 l/s was for U1 5%, from around 33% of the flow to 31%. Similar values were found when comparing the case with extension wall and corner profiles, resulting in a reduction of 5%. For only the corner profiles the reduction was 2%.

The main contribution to the larger differences in discharge distribution is concluded to be the extension wall, due to the restriction of inflow to a narrow channel. Another contributing factor is deemed to be the recirculation zone caused by the extension wall showing that the full width of the channel is not used to transport water from the inlet to the outlet. Further work in either experiments or CFD could explore the impact of the recirculation zone, and the flow conditions leading up to the channel as they impact the discharge capacity by large measurable quantities. Comparison of the calculated discharge coefficients show a linear behavior for the old case, while for all model changes which were investigated, a drop in discharge coefficient occurs as the flow increases. The configuration with altered corner profiles for the abutments saw a 3% reduction for discharge increasing from 60 l/s to 180 l/s. For the configuration with added extension wall a reduction of 3% was seen. For the configuration combining the two changes, a reduction of 5% was found. These drops occur at high levels of *h*/*P* greater than or equal to 1, which may have an impact on existing dams depending on their spillway design.

The clear difference in distribution of flow across the spillway for the case with the extension wall, together with the ADV-data can serve as excellent material for validation of CFD simulations. Successful implementations of the measurements as validation data would show how to perform simulations on multiple spillway setups so extrapolating the method to real world cases could be possible. This could lead to new solutions to design innovative and sustainable hydraulic structures. Other possible uses would be to simulate scale effects. The experimental setup can also serve as a basis for further experimental work in the area of multiple spillway outlets, with the possibilities of testing a combination of outlets, such as bottom outlets with different constructions and upstream flow.

## Data Availability

The experimental results obtained from the study can be acquired from the corresponding author upon request.
